# *Helicobacter pylori*-Induced Decrease in Membrane Expression of Na,K-ATPase Leads to Gastric Injury

**DOI:** 10.3390/biom14070772

**Published:** 2024-06-28

**Authors:** Olga Vagin, Elmira Tokhtaeva, Muriel Larauche, Joshua Davood, Elizabeth A. Marcus

**Affiliations:** 1 Department of Pediatrics, DGSOM at UCLA, 10833 LeConte Ave., 12-383 MDCC, Los Angeles, CA 90095, USA; olgav@ucla.edu (O.V.); elmiratim@ucla.edu (E.T.); 2 VA GLAHS 11301 Wilshire Blvd, Bldg 113, Rm 324, Los Angeles, CA 90073, USA; mlarauche@mednet.ucla.edu (M.L.); joshuadavood@g.ucla.edu (J.D.); 3 Department of Medicine, Vatche and Tamar Manoukian Division of Digestive Diseases, DGSOM at UCLA, 650 Charles E Young Dr. S., CHS 43-276, Los Angeles, CA 90095, USA

**Keywords:** *Helicobacter pylori*, Na,K-ATPase, ouabain, gastric injury, adherens junctions

## Abstract

*Helicobacter pylori* is a highly prevalent human gastric pathogen that causes gastritis, ulcer disease, and gastric cancer. It is not yet fully understood how *H. pylori* injures the gastric epithelium. The Na,K-ATPase, an essential transporter found in virtually all mammalian cells, has been shown to be important for maintaining the barrier function of lung and kidney epithelia. *H. pylori* decreases levels of Na,K-ATPase in the plasma membrane of gastric epithelial cells, and the aim of this study was to demonstrate that this reduction led to gastric injury by impairing the epithelial barrier. Similar to *H. pylori* infection, the inhibition of Na,K-ATPase with ouabain decreased transepithelial electrical resistance and increased paracellular permeability in cell monolayers of human gastric cultured cells, 2D human gastric organoids, and gastric epithelium isolated from gerbils. Similar effects were caused by a partial shRNA silencing of Na,K-ATPase in human gastric organoids. Both *H. pylori* infection and ouabain exposure disrupted organization of adherens junctions in human gastric epithelia as demonstrated by E-cadherin immunofluorescence. Functional and structural impairment of epithelial integrity with a decrease in Na,K-ATPase amount or activity provides evidence that the *H. pylori*-induced downregulation of Na,K-ATPase plays a role in the complex mechanism of gastric disease induced by the bacteria.

## 1. Introduction

About 50% of the world’s population is infected with *Helicobacter pylori* (*H. pylori*). The bacteria are able to colonize the normal acid-secreting stomach and infection leads to gastritis, gastric and duodenal ulcers, gastric carcinoma, and MALT lymphoma [1,2,3,4,5]. One hundred percent of those infected with *H. pylori* demonstrate histologic evidence of gastritis [6]. Chronic inflammation induced by the bacteria is a trigger for more advanced gastric disease, including potential development of gastric cancer [7]. *H. pylori* infection is the greatest known risk factor for gastric cancer and has a World Health Organization classification as a class I, or definite, carcinogen, with a 75% attributable risk [2,8]. *H. pylori* is also the most common cause of gastric and duodenal ulceration [5]. Gastric ulcers do not develop spontaneously in the normal stomach. Ulceration is a multifactorial process driven by the presence of acidity [9], and *H. pylori* renders the underlying serosa susceptible to acid-related damage. Successful treatment of *H. pylori* infection is effective in healing gastric injury, with >90% of those treated seeing ulcer resolution, and is effective in preventing bleeding recurrence [10,11,12]. It is not definitively known why some people infected with *H. pylori* develop gastritis while others develop more advanced disease. Increasing our understanding of the likely multifactorial process that allows for injury of the gastric epithelium by *H. pylori*, defined as a breakdown in barrier function, would be a critical step in moving the field closer to answering this question and in improving treatment protocols for advanced gastric disease.

Gastric acid is a critical protective agent in the stomach, with antimicrobial properties that have an impact across the length of the GI tract [13,14]. In the proper setting, epithelial injury can lead to damaging effects of acid, with risk of inflammation, ulcer, bleeding, or perforation [14,15,16]. When there is mucosal injury from acid, the epithelium is normally able to rapidly re-form cell junctions and regenerate epithelial cells [17]. *H. pylori* is able to upset this balance between acid injury and repair [18], but it remains unknown why this balance is upset in only some of those infected. With this in mind, a closer look at the gastric epithelial cell junction could provide more information regarding the mechanism of induction of gastric injury by *H. pylori*.

*H. pylori* bacteria have been shown to impair the epithelial barrier by targeting epithelial junctional complexes via direct interaction of *H. pylori* virulence factor CagA with cell adhesion proteins [19,20,21,22] and via CagA-dependent signal transduction pathways that target junctional complexes and cell polarity [23,24]. In addition, *H. pylori* bacteria impair cell adhesion by secreting virulence factor HtrA, which has been demonstrated to cleave the extracellular domains of tight junction proteins occludin and claudin and adherens junction protein E-cadherin [25,26]. Recent studies have identified the ion transport protein Na,K-ATPase as a cell adhesion molecule (reviewed in [27]), and *H. pylori* has been demonstrated to decrease Na,K-ATPase activity [28] and abundance [29], raising the question of whether this decrease contributes to the *H. pylori*-induced impairment of the gastric barrier. 

The Na,K-ATPase is found in the basolateral membrane of most mammalian cells. It uses the energy of ATP to move sodium out of cells and potassium into cells against their concentration gradients [30], generating ion gradients that are critical for membrane potential, transport of other ions, control of cell volume and pH, and nutrient uptake [31]. Maintenance of the ion gradients by the Na,K-ATPase is required for the formation and maintenance of intercellular junctions in epithelial cells [32,33,34,35,36]. In addition, the Na,K-ATPase also functions as a cell adhesion molecule independent of its transporter activity [37,38,39]. The β subunit connects to the β subunit in adjacent cells via trans-interactions between specific amino acid residues and N-glycans in the extracellular domain, while the α subunit links to the cytoskeleton, stabilizing this trans-bridge between β subunits and the junctional complex between cells [38,39,40,41]. Both the ion transport activity and the adhesive function of the Na,K-ATPase are independently and collectively important for the integrity of epithelial junctions [27,42].

A previous study demonstrated that *H. pylori* decreased ouabain-sensitive K^+^-dependent phosphatase activity in cultured gastric epithelial cells, suggesting that *H. pylori* targets Na,K-ATPase [28]. However, this study did not measure Na,K-ATPase abundance. We demonstrated that *H. pylori* infection of gastric epithelial cells led to decreased levels of Na,K-ATPase in the plasma membrane both acutely in cell culture models and chronically in infected gerbils and human patients [29]. When *H. pylori* directly attached to gastric epithelial cells, both surface and total levels of Na,K-ATPase were reduced. The mechanism of decrease in Na,K-ATPase by *H. pylori* was related to the degradation of newly synthesized subunits; no effect was seen on mRNA levels or on mature transporter protein already present in the plasma membrane. Infection with *H. pylori* specifically decreased the interaction of the ER chaperone BiP with Na,K-ATPase subunits, leading to the impaired assembly of subunits into mature transporter proteins and the ubiquitylation of the unassembled subunits, which targeted them for proteasomal degradation [29]. The resulting decrease in levels of plasma membrane Na,K-ATPase should therefore impair the ion gradients and weaken intercellular junctions, presenting a potential pathway by which *H. pylori* infection can lead to gastric injury. The aim of the current study was to demonstrate that a reduction in Na,K-ATPase in the plasma membrane of gastric cells causes gastric epithelial injury, providing insight into the mechanism of *H. pylori*-induced disease. 

## 2. Materials and Methods

### 2.1. Bacterial Strains and Culture Conditions

*H. pylori* strain G27, which is *cagA* and *vacA* positive [43,44], was used for all experiments. Prior to infection of cultured cells, *H. pylori* bacteria were grown on trypticase soy agar plates with 5% sheep blood (TSA plates) (ThermoFisher Scientific, Waltham, MA, USA) overnight in a mixed-gas incubator with 10% CO_2_ and 5% O_2_.

### 2.2. Cultured Cells

AGS (ATCC, Manassas, VA, USA) and HGE-20 cells [45] were grown in the mixture of equal volumes of Dulbecco’s modified Eagle medium (DMEM) (Cellgro Mediatech, Manassas, VA, USA) and F12 medium (Life Technologies, Carlsbad, CA, USA) with 10% FBS (Gemini Bio-Products, West Sacramento, CA, USA) and 100 units/mL penicillin, 0.1 mg/mL streptomycin (Sigma, St. Louis, MO, USA). HGE-20 cells were provided by Dr. Daniel Ménard, who kindly granted permission to use the cells for this work. HGE-20 cells form cell junctions and have polarized basolateral and apical plasma membrane domains [46].

### 2.3. Gastric Organoids

De-identified gastric tissue was obtained from the UCLA histopathology core via approved IRB exemption from post-operative gastric bypass samples. Briefly, gastric antrum tissue was washed in PBS before removing fat and connective tissue. It was then cut into ~1–2 mm pieces and washed in ice cold PBS until the supernatant was clear (8–10 times), followed by a 2 h incubation in chelating solution with 28 mM -Na_2_HPO_4_ (dibasic), 40 mM KH_2_PO_4_ (monobasic), 480 mM NaCl, 8 mM KCl, 220 mM sucrose; 274 mM d-sorbitol, 0.52 mM DTT added fresh and 10 mM EDTA, at a pH of 7.2–7.3 at 4 °C on a shaking platform. Tissue fragments were left to settle, then the chelating solution with EDTA was removed and 10 mL of chelating solution without EDTA and with 3% FBS was added to the tissue. Tissue was pipetted up and down 20–30 times, left to settle for 1 min, the supernatant was transferred to a new tube, and this process was repeated until enriched crypts were seen. Fractions were combined in a 50 mL tube, centrifuged for 5 min at 250× *g* at 4 °C, the supernatant was removed, and the pellet was re-suspended in 2 mL of advanced DMEM/F12 medium (ThermoFisher Scientific, Waltham, MA, USA). The number of crypts was estimated using a microscope, and re-suspended in an appropriate volume of Matrigel Matrix, Growth Factor Reduced Phenol Red-Free (Corning Inc., Corning, NY, USA). Approximately 50 crypts/25 µL Matrigel/well of a 48-well tissue culture plate were used to initiate 3D organoid cultures. Plates were incubated for at least 15 min in the 37 °C, 5% CO_2_ incubator to polymerize Matrigel. Then, 250 µL of the IntestiCult Organoid Growth Medium (Human) (StemCell Technologies, Kent, WA, USA) supplemented with 10 µM Rho kinase inhibitor Y27632 (Sigma, St. Louis, MO, USA) and 100 units/mL penicillin, 0.1 mg/mL streptomycin (Sigma, St. Louis, MO, USA) was added to each well and the plate returned to the incubator. The media were replaced with media without Y27632 after 2 days and were refreshed every 2–3 days.

### 2.4. Gastric Organoid Cells Grown as 2D Monolayers

Gastric organoids were harvested from 4 Matrigel domes, and suspensions were incubated in 1 mL of Gentle Cell Dissociation Reagent (StemCell Technologies, Kent, WA, USA) at room temperature for 10 min; then, suspensions were pipetted up and down using a 1 mL pipette to break up the Matrigel. TrypLE (ThermoFisher Scientific, Waltham, MA, USA) was added to dissociate organoids into individual cells, and after 2 min, the proteolysis was quenched by adding DMEM/F12 containing 10% FBS. Single-cell suspensions were plated into 6.5 mm diameter, 0.4 µm pore size Transwell™ inserts (Corning Inc., Corning, NY, USA) coated with a 1:50 diluted Matrigel solution. The cells were grown in IntestiCult Organoid Growth Medium containing 10 µM of Y27632 and 10 µM of Chir99021 (Sigma, St. Louis, MO, USA). Monolayers reached confluency within 3–5 days.

### 2.5. Silencing of Na,K-ATPase α_1_ Subunit

Lentiviral transduction of shRNA was used to reduce the expression of the Na,K-ATPase α_1_ subunit. The generation of lentiviral particles was performed in HEK 293T cells grown in DMEM, high-glucose, pyruvate medium (ThermoFisher Scientific, Waltham, MA, USA) containing 10% FBS (Gemini Bio-Products, West Sacramento, CA, USA), 100 units/mL penicillin, and 0.1 mg/mL streptomycin (Sigma, St. Louis, MO, USA) in 15 cm dishes. A solution containing 4 µg of pMD2.G VSV-G envelope expressing plasmid (gift from Didier Trono, Addgene plasmid #12259), 15 µg of pCMVR8.74 s generation lentiviral packaging plasmid (gift from Didier Trono, Addgene plasmid #22036), and 15 µg of 5 TRIPZ Inducible Lentiviral Human ATP1A1 shRNA vectors (Horizon Discovery. Cambridge, UK) in Opti-MEM medium (ThermoFisher Scientific, Waltham, MA, USA) was mixed with polyethylenimine (PEI) transfection reagent (Sigma, St. Louis, MO, USA). The mixture was incubated at room temperature for 10 min and carefully added dropwise to the cells. Sixteen hours after transfection, the media was replaced with 20 mL of growth media containing 20 mM N-2-hydroxyethylpiperazine-N-2-ethane sulfonic acid (HEPES) (ThermoFisher Scientific, Waltham, MA, USA) and 10 mM Na Butyrate (Sigma, St. Louis, MO, USA). Eight hours later, the media was replaced with 20 mL of growth media containing 20 mM HEPES. After 24 h, the culture medium was collected and replaced with fresh media. The collection of media was repeated every 24 h. The collected medium was stored at 4 °C. A total of 3 collections was made, combined media was filtrated through a (0.45 µm) filter, and the virus was concentrated by centrifugation at 100,000× *g* for 2 h at 4 °C in a continuous 0–20% sucrose gradient. The pellets (lentiviral particles) were re-suspended in 200 µL of the ice-cold sterile Hanks’ Balanced Salt Solution (HBSS) (ThermoFisher Scientific, Waltham, MA, USA) and stored at −80 °C until use. Confluent gastric organoid 2D monolayers grown in 6.5 mm diameter, 0.4 µm pore size Transwell™ inserts were infected with lentiviral particles using the following procedure. Lentivirus particle suspension was mixed with equal volume of IntestiCult Organoid Growth Medium (Human) (StemCell Technologies, Kent, WA, USA) supplemented with 10 µM Rho kinase inhibitor Y27632 and 10 µM Chir99021 (Sigma, St. Louis, MO, USA) and 100 units/mL penicillin, 0.1 mg/mL streptomycin (Sigma, St. Louis, MO, USA), and added to the cell monolayer. After a 24 h incubation of cell monolayers with lentivirus, the media containing lentivirus were removed and replaced with fresh organoid growth media containing 1.25 µg/mL pyromycin (InvivoGen, San Diego, CA, USA) to enrich the cells that were stably transduced by the lentivirus. The expression of TurboRFP/shRNAs was induced by adding 1 µg/mL doxycycline (Clontech Laboratories, Inc., Mountain View, CA, USA) to the cell monolayer, and TurboRFP fluorescence was monitored by fluorescent microscopy as the indication of shRNA expression. Cells were used on the 4th day after induction with doxycycline.

### 2.6. Infection of Gastric Cells and 2D Organoids with H. pylori

Gastric epithelial cells were grown on the appropriate inserts as indicated for specific experiments After reaching confluency, cell monolayers were washed with the medium without antibiotics and wells were infected from the apical side with *H. pylori* at an MOI of >100:1 to assure adherence. After 20 h of cell incubation with *H. pylori*, non-adherent bacteria were washed off. Cells were lysed, and total cell lysates were used as described for individual experiments.

### 2.7. Paracellular Permeability

The macromolecular paracellular permeability of 4 kDa fluorescein isothiocyanate-dextran, FD4, (Sigma-Aldrich, St. Louis, MO, USA) was measured across polarized monolayers of HGE-20 cells grown on 12 mm diameter, 0.4 µm pore size Snapwell™ inserts (Corning Inc., Corning, NY, USA). After reaching confluency, inserts were removed from wells and placed in between sliders (P2302) dedicated to the Ussing chamber (both sliders and the Ussing chamber were from Physiologic Instruments, Inc., Reno, NV, USA). The mucosal and serosal surfaces of the cell monolayers were bathed with 5 mL of oxygenated (carbogen) Krebs–Ringer solution containing (in mM) 115 NaCl, 1.2 CaCl_2_, 1.2 MgCl_2_, 4.8 KH_2_PO_4_, 48 K_2_HPO_4_, 25 NaHCO_3_, and 10 glucose and for experiments in the presence of ouabain, the indicated concentration of ouabain. The solution was bubbled with 95% O_2_–5% CO_2_ to maintain the pH at 7.4 and was kept at 37 °C during the course of the experiments by a circulating water bath heater. The cells were automatically short-circuited by a voltage clamp (Physiologic Instruments, Inc., Reno, NV, USA) at zero potential with compensation for solution resistance. After a stabilization period of 30–45 min as judged by a stable basal short-circuit current (*I*sc) and transepithelial electrical resistance (TEER) in the chambers, FD4 at a final concentration of 2.2 mg/mL was added to the apical compartment of the Ussing chamber. Every 30 min for 1.5 h of incubation at 37 °C, 50 μL of the basolateral solution was sampled, diluted 1:1 with PBS at pH 7.2, and fluorescence (Ex 485 nm; Em 525 nm) was measured using a microplate reader CLARIO star (BMG LABTECH Inc., Cary, NC, USA). Based on relative fluorescence units (RFUs), FD4 concentrations were calculated against a standard curve and expressed as a percentage of the FD4 concentration in untreated cells.

### 2.8. Trans-Epithelial Electrical Resistance (TEER) of Cell Monolayers

HGE-20 cells were grown on 24 mm diameter, 0.4 µm pore size Transwell™ inserts in 6-well plates (Corning Inc., Corning, NY, USA) or 2D organoids grown on 6.5 mm diameter, 0.4 µm pore size Transwell™ inserts (Corning Inc., Corning, NY, USA) for 7 days (for HGE-20 cells) and for 4 days (for 2D organoids) after reaching confluency. Cells were washed with a medium without antibiotics, and some wells were infected with *H. pylori* at an MOI of >100:1 to ensure adherence. After 16 h for HGE-20 cells or 20 h for 2D organoids, non-adherent bacteria were washed off with a DMEM:F12 medium. The TEER was measured in the absence or presence of *H. pylori* using the EVOM Epithelial Voltohmmeter and ENDOHM^®^-24 or EMDOM-6 chamber electrode set for EVOM (World Precision Instruments, Sarasota, FL, USA). Endohm^®^ chambers were placed in the microaerobic incubator with cords accessible from the outside so the incubator door would not be opened during the time course of the experiments. For experiments in the presence of ouabain, the medium containing the indicated ouabain concentration was added to both the upper and lower chambers. The TEER was measured as described above every 1 h for 4 h or as indicated. The average of 3 Transwell™ inserts per condition was calculated and expressed in Ohms×cm^2^.

### 2.9. TEER of Gerbil Gastric Tissue

All animal work was approved by the VA GLAHS IACUC. Male Mongolian gerbils were obtained from Charles River Laboratories (Wilmington, MA, USA). After appropriate quarantine, the gerbils were sacrificed. The gerbils were anesthetized with isoflurane 5% and decapitated. The stomach was opened along the greater curvature, and the lumen was gently washed in Krebs solution at room temperature. The stomach segments were pinned on a Sylgard-plated Petri dish containing ice-cold Krebs–Ringer solution (pH 7.4, 4 °C). Pyloric tissue was isolated from the whole stomach. The pylorus preparations of gerbil stomach were cut in 2 to 4 cross-sectional area sections (depending on the quality of the dissection) and mounted on 0.1 cm^2^ sliders (P2303) dedicated to the Ussing chamber (Physiologic Instruments, Inc., Reno, NV, USA). The mucosal and serosal surfaces of the tissue were bathed with 5 mL of oxygenated (carbogen) Krebs–Ringer solution containing (in mM) 115 NaCl, 1.2 CaCl_2_, 1.2 MgCl_2_, 4.8 KH_2_PO_4_, 48 K_2_HPO_4_, and 25 NaHCO_3_, with the addition of 10 mM glucose from the basolateral side and 10 mM mannitol from the apical side. The solutions were bubbled with 95% O_2_–5% CO_2_ to maintain the pH at 7.4 and kept at 37 °C during the course of the experiments by a circulating water bath heater. Tissues were left to equilibrate (as judged by a stable basal *I*sc and TEER) in the chambers for 45 min before conducting the experiments. The tissues were automatically short-circuited by a voltage clamp (Physiologic Instruments, Inc. Reno, NV, USA) at zero potential with compensation for solution resistance. *I*sc and tissue conductance (*G*t) were determined every 2 s and recorded by the DataQ system (Physiologic Instruments, Inc., Reno, NV, USA). Positive values for *I*sc indicate a negative electrical charge flux from the serosal→luminal bath, indicating anion secretion or cation absorption.

### 2.10. Immunofluorescence and Confocal Microscopy

Confluent HGE-20 cell monolayers grown on 24 mm diameter, 0.4 µm pore size Transwell™ porous inserts (Corning Inc., Corning, NY, USA) or 2D gastric organoids grown on 6.5 mm diameter, 0.4 µm pore size Transwell™ inserts (Corning Inc., Corning, NY, USA) were incubated with *H. pylori* or ouabain overnight as indicated for each experiment. The monolayer on the filters was then washed with PBS containing 1 mM MgCl_2_ and 1 mM CaCl_2_ twice and fixed with 4% paraformaldehyde for 15 min at 37 °C. After fixation, the porous membrane was cut using a sharp scalpel, transferred into a 24-well plate, and next steps were performed in the wells. For the staining of 3D gastric organoids, whole organoids were harvested and transferred into a 1.5 mL Eppendorf tube from a Matrigel dome using ice-cold PBS containing 1 mM MgCl_2_ and 1 mM CaCl_2_. The suspension was then pipetted up and down using a 1 mL pipette to break up the Matrigel and centrifuged for 20 s in a benchtop centrifuge. The supernatant was discarded, and the pellet was re-suspended in 4% PFA and incubated at 37 °C for 15 min for fixation. After fixation, organoids were washed with PBS. Permeabilization and blocking non-specific binding were performed by incubating HGE-20 cell monolayers or 3D organoids with the Dako Protein Block serum-free solution (Agilent Technologies, Inc., Santa Clara, CA, USA) containing 0.2% Triton for 30 min. Immunofluorescent staining was performed by a 1 h incubation with the primary antibodies against Na,K-ATPase α_1_ subunit Clone:C464.6 (EMD, Millipore, Temecula, CA, USA) and E-Cadherin Clone: 36 (BD Bioscience, Franklin Lakes, NJ, USA), followed by a 1 h incubation with Alexa Fluor 647- Goat anti-mouse antibodies (ThermoFisher Scientific, Waltham, MA, USA). The actin filaments were visualized using Alexa-Fluor-488-Phalloidin (ThermoFisher Scientific, Waltham, MA, USA) as described previously [39]. Stained cell monolayers on porous membranes or 3D organoids in suspension were mounted onto the slide. Confocal microscopy images were acquired using a Zeiss LSM 510 laser scanning confocal microscope (Carl Zeiss MicroImaging GmbH, Munich, Germany) and analyzed using ZEN Microscopy Software (Carl Zeiss MicroImaging GmbH, Munich, Germany). The total fluorescence signal on Na,K-ATPase α_1_ immunofluorescence images was collected by multiplying the mean fluorescence intensity by the area of the image. The total fluorescence measured and calculated the same way on the negative control images (organoids incubated without a primary antibody) was subtracted from a fluorescence intensity of experimental images. At least 5 images were quantified per condition for each biological repeat. The fluorescence signal of E-cadherin at the plasma membrane and inside the cells was compared by selecting regions of interest (ROIs) at the plasma membrane or in cytoplasm on E-cadherin immunofluorescence images and measuring the mean fluorescence intensity in each ROI. For each ROI at the plasma membrane or in cytoplasm, the control ROI with the same shape and area was selected in the region of the image without cells, and the mean fluorescence intensity of this control ROI was subtracted from the mean fluorescence intensity of the corresponding experimental ROI. The size of E cadherin clusters was analyzed by selecting ROIs for individual E-cadherin clusters and measuring their area. At least 20 ROIs per experimental condition were analyzed for each biological repeat.

### 2.11. Cell Lysis

After incubation at the indicated conditions, cells were washed twice with ice-cold PBS containing 1 mM MgCl_2_ and 1 mM CaCl_2_ on ice and lysed by incubating with 50 mM Tris, pH 7.5, containing 150 mM NaCl, 1% Nonidet P-40, 0.5% sodium deoxycholate and Complete Protease Inhibitor Cocktail, 1 tablet/50 mL, (Roche Diagnostics, Indianapolis, IN, USA) at 4 °C for 30 min. Cells were scraped from the plates or Transwell™ inserts, and insoluble material was removed by centrifugation (20,000× *g*, 15 min) at 4 °C.

### 2.12. Western Blotting

Proteins in cell lysates were size-fractionated by SDS-PAGE and transferred to nitrocellulose membranes for Western blotting. Primary antibodies against Na,K-ATPase α_1_ subunits (1:1000; mouse, Clone:C464.6, EMD, Millipore, Temecula, CA, USA) and Na-K-ATPase β1-subunits (1:1000; mouse, clone M17-P5-F11, Affinity Bioreagents, Golden, CO, USA) were used to detect Na,K-ATPase. Primary antibodies against NSF, N-ethylmaleimide-sensitive factor (1:1000; mouse, clone NSF-1, EMD, Millipore, Temecula, CA, USA), and β-actin (1:1000; rabbit, Cell Signaling, Danvers, MA, USA) were used for loading controls. Secondary antibodies were horseradish peroxidase-linked goat anti-mouse or goat anti-rabbit (1:10,000; American Qualex, San Clemente, CA, USA). Bands were detected using a SuperSignal West Pico Chemiluminescence Kit (Thermo). Immunoblots were quantified by densitometry using Image Studio Software (LI-COR, Lincoln, NE, USA).

## 3. Results

### 3.1. H. pylori Infection and Inhibition of Na,K-ATPase Activity with Ouabain Impair Intracellular Junctions in Cultured Gastric Epithelial Cells and Gerbil Gastric Mucosa

**Figure 1:**
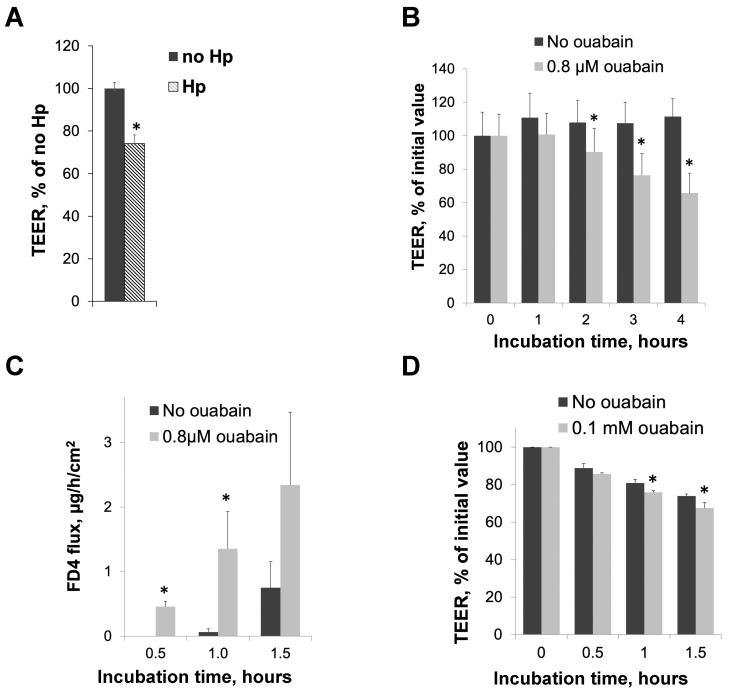
Both *Helicobacter pylori* infection and reduced Na,K-ATPase activity impair intracellular junctions in cultured cells (**A**–**C**) and in gerbil mucosa (**D**). HGE-20 cells were grown to confluency in Transwell™ inserts, and the transepithelial electrical resistance (TEER) was measured in the presence of *H. pylori* (**A**) or ouabain (**B**). The TEER is expressed as a percentage of the initial value. Initial values of the TEER in HGE-20 cells ranged from 1000 to 1500 Ω×cm^2^. Paracellular permeability of confluent HGE-20 cell layers was measured in an Ussing chamber in the presence or absence of ouabain, results are expressed as the rate of FD4 flux through the cell layer (**C**). Gerbils were euthanized and stomach tissue incubated in an Ussing chamber in the presence of ouabain, and the TEER was measured at the indicated times. Results are expressed as percent of initial value (**D**). Initial values of the TEER in gerbil gastric mucosa ranged from 75 to 100 Ω×cm^2^, consistent with prior published results [57]. For all panels, *n* = 3, * *p* < 0.05 (*t*-test), mean ± SD.

HGE-20 gastric epithelial cells were incubated with *H. pylori* or a vehicle for 16 h, then resistance was measured in an Endohm^®^ chamber. There was a significant reduction in TEER in the presence of *H. pylori* at 16 h (Figure 1A), suggestive of injury to the gastric barrier. The experiment was repeated in the presence of ouabain, an inhibitor of the Na,K-ATPase [47,48], instead of *H. pylori* to determine if a direct inhibition of the Na,K-ATPase also led to decreased barrier function. We chose the concentrations of ouabain that effectively inhibited the Na,K-ATPase activity in different model systems based on our preliminary data on ouabain’s effect on ion transport activity of human Na,K-ATPase [49] and on the literature on ouabain sensitivity in rodents [50,51,52,53,54,55,56]. There was a significant drop in TEER starting at 2 h at the higher concentration of ouabain and starting at 3 h for both measured concentrations, as compared to the vehicle (Figure 1B, higher concentration shown). The longer time course was needed for the bacterial experiments because the bacteria needed time to attach and divide, while a shorter time course was needed for ouabain to ensure the stability of the chemical. Both *H. pylori* and ouabain interfered with barrier function. Paracellular permeability was measured by FD4 flux through the cell layer in an Ussing chamber, in the presence of ouabain or the vehicle. The presence of ouabain led to an increase in paracellular permeability compared with the control, again suggesting compromised barrier function (Figure 1C). This is in agreement with previous results demonstrating increased paracellular permeability in HGE-20 gastric epithelial cells in the presence of *H. pylori* [46]. Gerbils were sacrificed and stomach sections were placed in the Ussing chamber in the presence of ouabain at 0.1 mM. The higher concentration is required for gerbil tissue due to the lower Na,K-ATPase sensitivity to ouabain in rodents [50,51,52,53,54,55,56]. There was a significant drop in TEER in the presence of ouabain after 1 h of incubation as compared with the vehicle, confirming that the inhibition of the Na,K-ATPase also impairs barrier function in vivo (Figure 1D). Relatively low values for the TEER in gerbils are in agreement with previously published results [57].

### 3.2. Human Gastric Organoids Express Na,K-ATPase and Demonstrate Reduction in Transporter in the Membrane in the Presence of H. pylori

**Figure 2:**
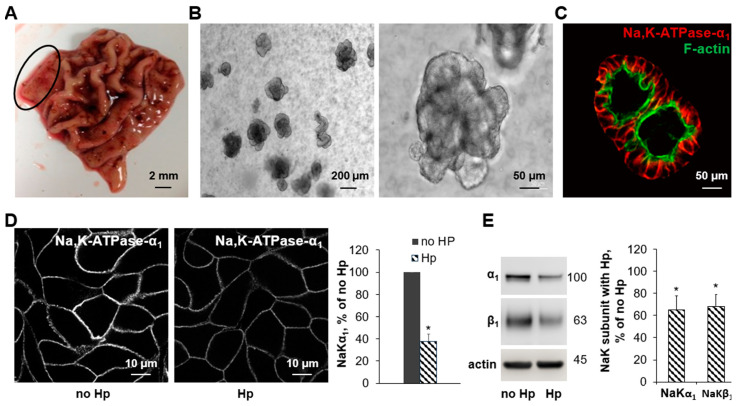
Human gastric organoids as a model system to study the effect of *Helicobacter pylori* on gastric mucosa. Human gastric antrum was obtained from de-identified sleeve gastrectomy specimens. The location of the harvested sample is indicated by the circle (**A**). Human gastric organoids were cultivated from the fresh tissue samples and plated in Matrigel with appropriate growth factors, leading to the formation of 3D organoids (**B**). Basolateral expression of Na,K-ATPase was confirmed in gastric organoids by immunofluorescence of the α_1_ subunit, indicated by the red color, and F-actin was used as a counterstain, indicated by the green color (**C**). Three-dimensional organoids were converted to 2D using Matrigel-coated Transwell™ inserts and were grown into a confluent monolayer. Organoids were incubated with the vehicle or *H. pylori* (**D**,**E**), and the decrease in Na,K-ATPase subunits in the presence of *H. pylori* was confirmed in this model system by immunofluorescence (**D**) or Western blot (**E**). Quantification is shown in the right panels. Mean ± SD, *n* = 3, * *p* < 0.05, *t*-test.

Gastric glands were isolated from de-identified sleeve gastrectomy samples, obtained at UCLA via IRB exemption. The gastric antrum was removed from the edge of the sample (see circled area in Figure 2A), glands were isolated and plated in Matrigel with growth factors. Three-dimensional gastric organoids grew in the Matrigel (Figure 2B) and the expression of the Na,K-ATPase in the membranes of these cells was confirmed by immunofluorescence with F-actin as a counterstain (Figure 2C). The Na,K-ATPase was expressed in the basolateral membranes of the gastric cells as expected. Three-dimensional organoids were successfully converted to 2D layers using Matrigel-coated Transwell™ inserts, and the TEER was noted to increase with time in culture, with the resistance of the confluent monolayer measured at an average of 1342 ± 45 Ω×cm^2^. Infection of 2D gastric organoids with *H. pylori* resulted in a significant decrease in Na,K-ATPase as shown by immunofluorescence with antibodies against the Na,K-ATPase α subunit (Figure 2D) and by Western blot using antibodies against the α and β subunits (Figure 2E). Two-dimensional gastric organoids are a viable model system for studying the effect of *H. pylori* on Na,K-ATPase levels.

### 3.3. H. pylori Infection, Na,K-ATPase Silencing, and Inhibition of Na,K-ATPase Activity All Decrease TEER in Human Gastric Organoids

**Figure 3:**
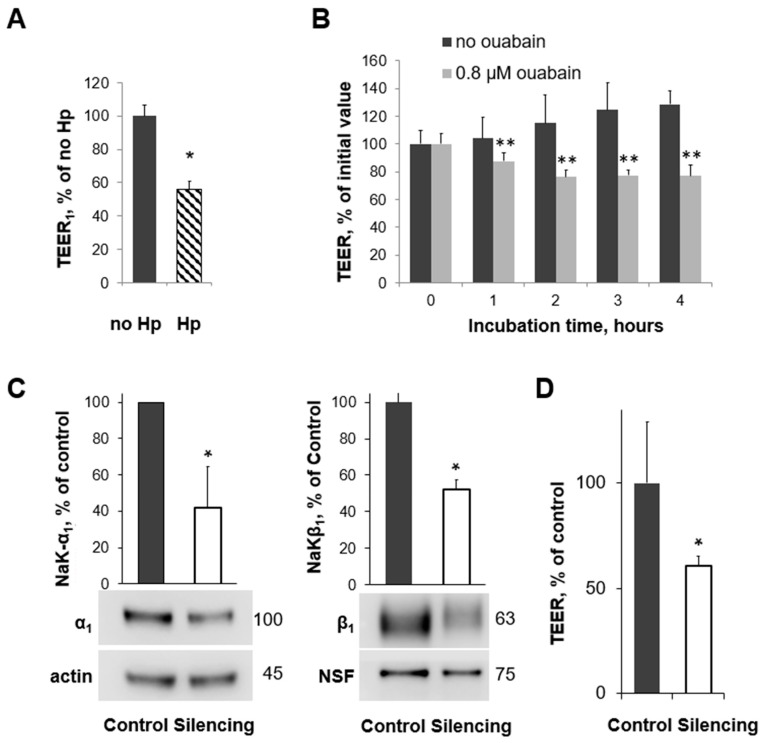
*Helicobacter pylori* infection, reduced amount of Na,K-ATPase, and reduced Na,K-ATPase activity similarly decrease the TEER in human gastric organoids. (**A**,**B**) The TEER of 2D human gastric organoids grown on Transwell™ inserts and measured in an Endohm^®^ chamber was decreased after exposure to *Helicobacter pylori* (**A**) and in the presence of ouabain compared with the vehicle (**B**). Western blot showing the silencing of the Na,K-ATPase α_1_ subunit (**C**). The TEER of 2D human gastric organoids grown on Transwell™ inserts and measured in an Endohm^®^ chamber was decreased after silencing the Na,K-ATPase α_1_ subunit (**D**). Initial values of the TEER in 2D gastric organoids ranged from 800 to 1300 Ω×cm^2^. Mean ± SD, significant differences from control, *t*-test, *n* = 3, * *p* < 0.05 (**A**,**C**,**D**) and n = 5, ** *p* < 0.01 (**B**).

The TEER of 2D human gastric organoids grown on Transwell™ inserts and measured with an Endohm^®^ chamber decreased to about 55% of that of the control when infected with *H. pylori* for 20 h (Figure 3A). Experiments were repeated in the presence or absence of ouabain, which inhibits the function of the Na,K-ATPase. A four-hour time course of TEER measurements is shown in Figure 3B. As expected, the effect is more rapid with ouabain, and it is sustained. The inhibition of Na,K-ATPase function with ouabain leads to a reduction in TEER compared with the vehicle (Figure 3B) to a similar degree as is seen with *H. pylori* infection. This suggests that the number of transporters present and the transporter function are both important for the prevention of gastric injury. Based on these TEER measurements, 2D human gastric organoids showed similar impairment in cell junctions when Na,K-ATPase activity was inhibited and when infected with *H. pylori*. Next, a partial silencing of the Na,K-ATPase α_1_ subunit was completed using a lentivirus transduction of shRNA, and expression was decreased to about 40% of the control levels once the construct was induced (Figure 3C). Full silencing would not allow for cell survival. The TEER was measured in an Endohm^®^ chamber once the Na,K-ATPase α_1_ subunit was decreased and was significantly reduced compared with that of the control (Figure 3D). Decreased expression, chemical inhibition, and downregulation for the Na,K-ATPase by *H. pylori* all contributed to a similar decrease in TEER in gastric organoids, confirming the importance of the Na,K-ATPase in the barrier function of gastric cells and providing evidence of a potential mechanism for *H. pylori* to contribute to gastric injury.

### 3.4. Reduction of Na,K-ATPase Activity with Ouabain or Downregulation with H. pylori Disrupts the Organization and Integrity of Adherens Junctions in 2D Human Gastric Organoids

**Figure 4:**
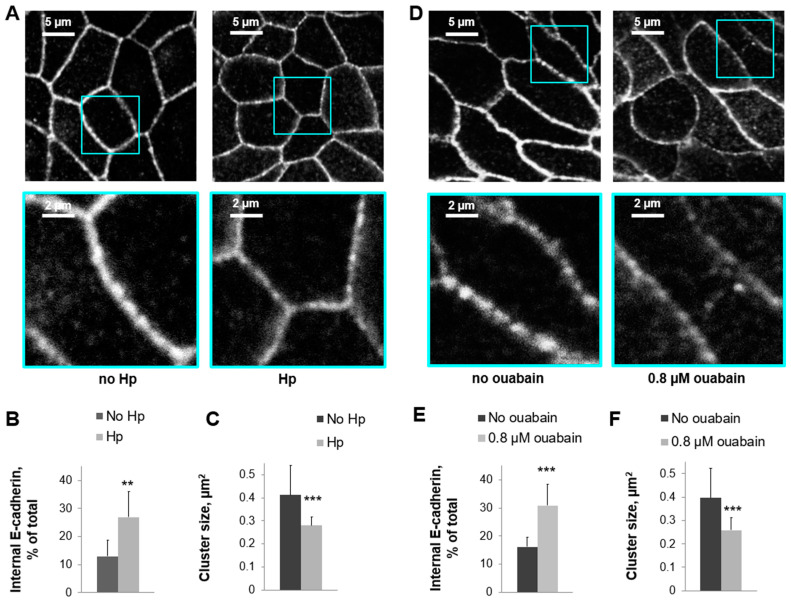
Reduced Na,K-ATPase activity and *Helicobacter* infection similarly impair adherens junctions in human gastric organoids. Immunofluorescence of 2D gastric organoids using E-cadherin antibodies showed a decreased E-cadherin signal at the cell junctions in the presence of *H. pylori* (**A**) or ouabain (**D**) and an increased intracellular accumulation of E-cadherin (**B**,**E**). E-cadherin clusters were decreased in size in the presence of *H. pylori* (**C**) or ouabain (**F**), suggesting injury to junctions. Mean ± SD, significant differences from “no Hp” or “no ouabain”, *t*-test. *n* = 5, ** *p* < 0.01, *** *p* < 0.001 (**B**,**F**).

E-cadherin is a critical component of the adherens junctions between epithelial cells. Immunofluorescence using antibodies against E-cadherin was completed on 2D gastric organoids in the presence or absence of *H. pylori*. The E-cadherin signal in cell junctions was decreased, while the intracellular staining was increased in the presence of *H. pylori* (Figure 4A–C). In addition, *H. pylori* decreased the size of E-cadherin clusters (Figure 4C), which are known to be formed in tight epithelial monolayers [58]. These experiments were repeated in the presence of ouabain instead of *H. pylori*, and the findings were similar. Incubation with ouabain led to the increased internalization of E-cadherin in 2D gastric organoids (Figure 4D–F) and a decrease in the size of E-cadherin clusters in the cell junctions (Figure 4F). Changes in the organization of E-cadherin suggests a disruption of the adherens junctions both when the Na,K-ATPase is chemically inhibited or downregulated by *H. pylori*. This provides further functional and structural evidence of gastric injury.

## 4. Discussion

*H. pylori* infection is highly prevalent and causes a disease burden ranging from gastritis to gastric cancer. Despite variable progression to advanced disease such as ulcers or gastric cancer, infection with *H. pylori* uniformly starts with inflammation and injury to the gastric epithelial barrier. *H. pylori* attachment to gastric cells in acute and chronic infection models leads to the decreased assembly of newly made Na,K-ATPase subunits and decreased delivery of new pumps to the plasma membrane [29]. The Na,K-ATPase is known to be important for the integrity of intracellular junctions in epithelia [36,39,59], and both Na,K-ATPase transport activity and the direct role of the Na,K-ATPase as a cell adhesion molecule in making intercellular bridges contribute to the role of the enzyme in maintaining barrier function. The important question that arises from these findings is if the downregulation of the Na,K-ATPase in the presence of *H. pylori* leads to gastric injury by compromising barrier function.

This question was addressed by studying the effects of a partial inhibition of the Na,K-ATPase activity and by the partial silencing of the Na,K-ATPase on gastric barrier function in various model systems, including cultured gastric cells, human gastric organoids, and gastric epithelium isolated from gerbils. It has been shown in the past and is re-demonstrated in Figure 1A that the incubation of cultured gastric epithelial cells in the presence of *H. pylori* leads to a decrease in TEER [60]. When the same cultured cells are incubated with ouabain, a specific and direct inhibitor of the Na,K-ATPase [61], the TEER also decreases, demonstrating the role of the transport activity of the Na,K-ATPase in barrier function (Figure 1B). Consistent with these data, ouabain increased the mucosal-to-serosal flux of FD4, a fluorescent dye, representing the paracellular permeability of the epithelial cell monolayer, as shown in Figure 1C. More dye is able to move between cells in the presence of ouabain than without, which when considered along with the reduction in TEER suggests a compromised barrier when there is less Na,K-ATPase activity. Importantly, ouabain has a similar effect on intact epithelium from gerbils (Figure 1D), with a reduction in TEER over time. The gerbil stomach is a reliable model for studying the epithelial injury associated with *H. pylori*-induced gastric disease [62], so confirming the effect of the decreased Na,K-ATPase activity in intact gerbil tissue provides further support for the relevance of the effect of *H. pylori* on Na,K-ATPase.

Silencing the Na,K-ATPase in gastric tissue to provide a physical reduction in the membrane presence of the transporter would be technically difficult in the gerbil model. To overcome this challenge and still utilize a model system that would recapitulate the epithelium of the stomach, human gastric organoids were adapted to further study the effect of both chemical and physical inhibitions of the Na,K-ATPase function. We were able to grow organoids from human gastric antrum samples and confirmed the expression of the Na,K-ATPase protein in the basolateral membrane by immunohistochemistry (Figure 2A–C). Since 3D organoid structures have an apical inside conformation and there are technical challenges with measuring the TEER, it was critical to be able to adapt a 2D model system for comparative studies. Once the cells were successfully grown in a monolayer, the epithelium was shown to be tight, and *H. pylori* induced a decrease in Na,K-ATPase that was consistent with what was demonstrated in cultured cells (Figure 2D,E). Once it was demonstrated that 2D gastric organoids had a similar response to *H. pylori* infection and ouabain (Figure 3A,B), inducible silencing was employed to determine the effect of a physical decrease in Na,K-ATPase on epithelial integrity, independent of *H. pylori* infection. Inducible silencing was necessary since Na,K-ATPase activity is required for cell function [31]. This allowed a reduction in the transporter amount similar to what is seen in the presence of *H. pylori* while ensuring continued cell layer viability (Figure 3C). Silencing the Na,K-ATPase led to a similar reduction in TEER as compared with ouabain and *H. pylori* infection (Figure 3D). Taken together, the impairment of epithelial integrity via three different mechanisms of decreasing the Na,K-ATPase amount or activity provides evidence for the role of the transporter in gastric epithelial barrier function and supports a link between the effect of *H. pylori* on Na,K-ATPase and *H. pylori*-induced gastric injury.

Epithelial junction complexes include tight junctions and adherens junctions and function to maintain the integrity of the epithelial barrier [63]. The Na,K-ATPase is precisely co-localized with E-cadherin at the adherens junctions [34,39,42,59]. Several experiments have demonstrated a functional link between the Na,K-ATPase and E-cadherin. The stability of E-cadherin-mediated junctions, as accessed by the detergent resistance of E-cadherin, was decreased by the removal of N-glycans from the β_1_ subunit [39]. Further, the overexpression of E-cadherin and the Na-K-ATPase β_1_ subunit in MSV-MDCK cells, which are not able to form functional tight junctions, increased the resistance of E-cadherin to detergent extraction and facilitated the formation of tight junctions [37]. In contrast, the overexpression of E-cadherin alone did not reestablish intercellular junctions [37]. These results indicate that the presence of the Na,K-ATPase β_1_ subunit is required for a stable association of E-cadherin with the cytoskeleton. It was shown using super-resolution microscopy techniques that E-cadherin specifically clusters within the adherens junctions and rapidly and constantly rearranges, allowing for the stability of the region over time [58]. E-cadherin outside of the adherens junctions is monomeric [58]. The cluster size of E-cadherin that reflects the strength of adherens junctions [64] was decreased in the presence of ouabain in gastric organoids as detected using immunofluorescence (Figure 4). In parallel, more E-cadherin was detected inside the cells, indicating the disassembly of E-cadherin complexes at the adherens junctions due to the lack of Na,K-ATPase activity. Similar effects of *H. pylori* on E-cadherin distribution strongly suggest the contribution of the bacteria-induced decrease in Na,K-ATPase.

Our data on the impairment of gastric epithelial barrier by ouabain are consistent with previously published results in epithelial cells in culture. Incubation of various epithelial cells with ouabain prevented tight junction formation [34,36], triggered the disassembly of existing junctions [32,33], and increased their permeability [35]. In most cases, the ouabain-dependent effects on cell adhesion were similar to the effects seen at low K^+^ concentration or at high Na^+^ concentration [32,36], demonstrating that the maintenance of proper ion gradients by the Na,K-ATPase is crucial for intercellular junctions. However, the impairment of epithelial junctions can also result from the inhibition of the adhesive function of the Na,K-ATPase. Particularly, mutations that prevent the N-glycosylation of the β_1_ subunit impair the formation of β_1_–β_1_ bridges, decrease the stability of adherens junctions and increase paracellular permeability in cultured cells [39,40,41] but has no effect on the α_1_β_1_ assembly [39,65,66], on the number of Na,K-ATPase molecules at the plasma membrane, or on Na,K-ATPase activity [39,65,66,67,68,69].

Furthermore, epithelial barrier injury due to a decrease in Na,K-ATPase activity or amount has also been demonstrated in vivo. In the alveolar epithelium of the lung, hypoxia triggers the endocytosis of the Na,K-ATPase [70]. The alveolar epithelium is critical for fluid reabsorption, and alterations in ion flux in response to the decreased availability of the Na,K-ATPase leads to impaired fluid clearance and increased pulmonary edema [71]. The overexpression of the Na,K-ATPase in rat models of lung injury and normal lung leads to increased fluid resorption, which may provide a future therapeutic pathway [72,73,74]. During blastocyst formation in early mouse embryos, ouabain treatment, K+ depletion, or knockdown of the Na,K-ATPase β_1_ subunit disrupted the normal formation of tight junctions and inhibited blastocyst formation [75,76]. In Drosophila, the Na,K-ATPase is required for the formation of septate junctions (reviewed in Ref. [77]). During the embryonic development of zebra fish, the Na,K-ATPase is required for heart morphogenesis and brain ventricle formation [78,79,80]. The inhibition of transport activity of the Na,K-ATPase by ouabain or by mutations resulted in a loss of ZO-1 junction belts in the developing heart, indicating that the Na,K-ATPase and correct ionic balance of myocardial cells are essential for the maintenance of cell junctions during heart morphogenesis [81]. Similarly, in Drosophila, the Na,K-ATPase is required for the formation of septate junctions during development, as was demonstrated by analyzing Na,K-ATPase mutants (reviewed in Ref. [77]).

A decrease in Na,K-ATPase activity additionally leads to cell injury as a consequence of downstream changes such as alterations in cell volume or secondary active transport. In a study conducted shortly after the discovery of *H. pylori*, a significant correlation was reported between epithelial cells heavily infected with the bacteria as seen on endoscopy and the presence of gastritis. Ultrastructural changes in epithelial cells heavily infected with bacteria were noted, most significantly, cellular edema [82]. These effects of *H. pylori* would be consistent with decreased Na,K-ATPase activity since decreased movement of sodium out of the cell causes more water to move in. The concentration gradient established by normal Na,K-ATPase activity is critical for nutrient uptake. Reduced Na,K-ATPase activity in skeletal muscle of growth-restricted fetal sheep leads to ecreased uptake of amino acids via sodium-dependent transporters, which may lead to a persistent reduction in muscle mass and long-term health concerns [83]. Sodium chloride and glucose homeostasis in the intestine are dysregulated in hypertension and diabetes seen with obesity, and alterations in the activity of the Na,K-ATPase in the intestinal villi may be the initial step in this process, as evidenced by studies in rat models, human cells, and human intestinal biopsies [84]. These studies emphasize the critical nature of Na,K-ATPase activity in normal cellular processes in various organ systems and the cell injury that can result when activity is altered. Recent studies suggest that the Na,K-ATPase, in addition to its roles in ion transport and cell adhesion, participates in various signaling pathways initiated by endogenous cardiotonic glycoside-like compounds, such as marinobufagenin [85,86]. Future studies will address the question of whether the decrease in Na,K-ATPase by *H. pylori* affects these signaling pathways.

The Na,K-ATPase is critical for the generation of ion gradients and for cell adhesion. Both inhibition and silencing of Na,K-ATPase specifically impair barrier function in gastric cells, indicating the importance of Na,K-ATPase in the maintenance of gastric epithelial junctions. Since *H. pylori* induces a reduction in plasma membrane Na,K-ATPase abundance and activity, this decrease, along with the known effects of *H. pylori* virulence factors CagA and HtrA, must contribute to the damaging effect of bacteria on the epithelial barrier, potentially initiating or aiding in the cascade of events that lead to the inflammation and erosion of the protective barrier in the stomach. These findings provide insight into the multi-factorial mechanism of gastric injury by the bacteria and provide a novel target for the management or mitigation of the consequences of *H. pylori* infection.

## Data Availability

The original contributions presented in the study are included in the article, further inquiries can be directed to the corresponding author.
